# Vomitophobia in atypical anorexia nervosa

**DOI:** 10.1192/j.eurpsy.2021.1855

**Published:** 2021-08-13

**Authors:** A. Bryukhin, T. Lineva, E. Okonishnikova

**Affiliations:** Department Of Psychiatry And Medical Psychology, RUDN University Moscow., Moscow, Russian Federation

**Keywords:** eating disorder, Vomitophobia

## Abstract

**Introduction:**

In atypical anorexia nervosa, one of the causes of restrictive eating behavior is prolonged vomitophobia, which leads to a pronounced degree of alimentary exhaustion.

**Objectives:**

To study the existence and prevalence of vomitorium atypical anorexia nervosa

**Methods:**

Psychopathological, anamnestic, psychological

**Results:**

It was found that in atypical anorexia nervosa, vomitophobia is observed in 30% of cases. The initial stage is a psychotraumatic situation unrelated to eating behavior. In the future, the pathological fear of vomiting is fixed, which is due to the presence of personal deviations and anxiety disorders. The initial stage of an eating disorder is a psychotraumatic situation that is not directly related to eating behavior. However, after Psychotrauma, there is anxiety with its subsequent somatization and vegetative dysfunction of the gastrointestinal tract. In the future, the pathological fear of nausea and vomiting is fixed, which is due to the presence of significant personal characteristics and persesting of anxiety disorders. Dysmorphophobic experiences appear as you lose weight and have an inverted character-discontent with thinness, exhaustion.

**Conclusions:**

The presence of massive vomitorium leads to restrictive eating behavior. Therefore, there is a need to differentiate this pathology from typical anorexia nervosa with vomiting and from hypochondriac disorders. Against the background of adequate complex therapy with food rehabilitation, anti-anxiety medication, psychotherapy, atypical anorexia nervosa with vomitophobia undergoes a fairly rapid reverse dynamics with the appearance of a critical attitude to the existing pathological eating behavior. The prognosis in these cases is quite favorable.

**Disclosure:**

No significant relationships.

